# Adherence to a priori dietary patterns in relation to obesity: results from two cycles of the Canadian National Nutrition Survey

**DOI:** 10.1017/S1368980023000903

**Published:** 2023-08

**Authors:** Alena Praneet Ng, Mahsa Jessri, Mary R L’Abbé

**Affiliations:** 1 Department of Nutritional Sciences, Faculty of Medicine, University of Toronto, Toronto, ON, Canada; 2 Food, Nutrition and Health Program, Faculty of Land and Food Systems, The University of British Columbia, Vancouver, BC, Canada; 3 Centre for Health Services and Policy Research (CHSPR), Faculty of Medicine, The University of British Columbia, Vancouver, BC, Canada

**Keywords:** National Cancer Institute method, Dietary Approaches to Stop Hypertension, Dietary Guidelines for Americans Adherence Index, Mediterranean-Style Dietary Pattern Score, Canadian Community Health Survey

## Abstract

**Objective:**

To test whether adherence to the Mediterranean diet, the Dietary Approaches to Stop Hypertension (DASH) or a dietary pattern in-line with the 2015–2020 Dietary Guidelines for Americans (DGA) was associated with obesity.

**Design:**

24-h dietary recall data from the Canadian Community Health Survey (CCHS)-Nutrition, 2004 and 2015 cycles, were analysed. Diet quality index scores were computed for the Mediterranean-Style Dietary Pattern Score (MSDPS), a DASH index and the 2015 Dietary Guidelines for Americans Adherence Index (DGAI). Higher scores indicated greater adherence. Association between scores and obesity was examined using logistic regression, adjusting for age, sex, physical activity, smoking status, sequence of dietary recall and alcohol and energy intake.

**Setting:**

Canada (excluding territories and the institutionalised population).

**Participants:**

Canadian adults (≥ 18 years), non-pregnant and non-breast-feeding; 11 748 from CCHS 2004 and 12 110 from CCHS 2015. The percentage of females in each sample was 50 %.

**Results:**

Mean MSDPS, DASH and DGAI scores were marginally but significantly higher in CCHS 2015 than in CCHS 2004. Those affected by obesity obtained lower scores for all indexes in CCHS 2004 (OR 10th *v*. 90th percentile for DASH: 2·23 (95 % CI 1·50, 3·32), DGAI: 3·01 (95 % CI 1·98, 4·57), MSDPS: 2·02 (95 % CI 1·14, 3·58)). Similar results were observed in CCHS 2015; however, results for MSDPS were not significant (OR 10th *v*. 90th percentile for DASH: 2·45 (95 % CI 1·72, 3·49), DGAI: 2·73 (95 % CI 1·85, 4·03); MSDPS: 1·30 (95 % CI 0·82, 2·06)).

**Conclusion:**

Following DASH or the 2015–2020 DGA was associated with a lower likelihood of obesity. Findings do not indicate causation, as the data are cross-sectional.

There has been a focus on the role of dietary patterns in obesity aetiology in recent years^(1,2)^. The Mediterranean dietary pattern, with its focus on whole fruits, vegetables and olive oil, may be protective against long-term weight gain^(3–6)^ and incident obesity^(6)^. The Dietary Approaches to Stop Hypertension (DASH) dietary pattern, high in Ca and K-rich foods such as fruits and vegetables, has been associated with significantly lower weight gain in adolescents^(7,8)^ and adults^(9)^. The 2015 Dietary Guidelines for Americans Adherence Index (DGAI), which measures adherence to the 12 USDA Food Patterns for chronic disease prevention described in the 2015–2020 Dietary Guidelines for Americans (DGA)^(10,11)^, has been cross-sectionally associated with a lower likelihood of obesity among Canadian adults^(10,12)^ and in the SU.VI.MAX French cohort^(4)^.

Many of the studies applying comparisons to multiple dietary patterns using rigorous, evidence-based diet quality indexes are based in the USA and utilise prospective cohort data. There is a lack of research that exists on the role of healthy dietary patterns and the likelihood of being affected by obesity utilising nationally representative health and nutrition survey data, particularly in the Canadian context, and specifically for promising dietary patterns for chronic disease prevention such as DASH and the Mediterranean diet^(10,12,13)^. This is a crucial knowledge gap, as an estimated one in four Canadian adults are currently living with obesity and an estimated one in thirty are living with multiple chronic diseases^(14)^.

The objectives of this study were to apply three diet quality indexes – the 2015 DGAI^(10)^, the Mediterranean-Style Dietary Pattern Score (MSDPS)^(15)^ and a DASH index^(16)^ to two cycles of nationally representative Canadian health and nutrition survey data in order to examine the relationship between adherence to healthy dietary patterns and concurrent likelihood of being affected by obesity.

Studies utilising a nationally representative Canadian population can offer international audiences insight into how various dietary patterns perform in varied settings and offer a distinct population for global researchers to compare study findings across public health benchmarks. Furthermore, this study provides key methodological considerations and insights into the application of diet quality indexes in the context of national health and nutrition survey data, including application of the National Cancer Institute (NCI) method for estimating usual intakes using repeat 24-h dietary recall data and applying validated correction factors to partially account for systemic biases introduced in self-reported BMI values.

## Subjects and methods

### Study population and data collection

Data for this study were collected under the authority of the Statistics Act of Canada. Analyses were conducted at the Statistics Canada’s Research Data Center in Toronto, Canada. All data were de-identified and accessible through Statistics Canada’s Research Data Centers.

Cross-sectional data were taken from 2004 and 2015 collections of the Canadian Community Health Survey (CCHS)-Nutrition, which provided the most-recent, nationally representative data available for Canadians’ food and beverage intakes^(17)^. The sample frame included Canadians ≥ 0 year for CCHS 2004 and ≥ 1 year for CCHS 2015; members of the Canadian Forces and those living in the territories, on reserves, in prisons or in long-term care facilities were excluded from both cycles. In CCHS 2004, 35 107 respondents were sampled and weighted by Statistics Canada to represent 98 % of the Canadian population with a survey response rate of 76 %; in CCHS 2015, 20 487 respondents were sampled with a response rate of 62 %.

In both cycles, one respondent per household was selected to complete a general health questionnaire; anthropometric measurements were also taken with respondent consent. One 24-h recall was completed with the assistance of a trained Statistics Canada interviewer using a computerised, Canadian modification of the USDA’s Automated Multiple Pass Method^(18)^; recall interviews were conducted from January 2015 to December 2015 during all 7 d of the week. Approximately 35 % of respondents completed a second recall by phone 7–10 d later. The nutrient and energy composition of consumed foods were obtained from the Canadian Nutrient File, Supplement 2001b^(19)^ and 2015^(20)^ for CCHS 2004 and CCHS 2015, respectively.

Additional details on CCHS-Nutrition survey design and the dietary recall component can be accessed elsewhere^(17,18)^.

### Outcome measures and exclusion criteria

The primary outcome of interest was BMI ≥ 30 kg/m^2^.

CCHS 2004 data were accessed, and all analyses completed in Summer 2017. Exclusion criteria included: respondents < 18 years, pregnant or breast-feeding women, underweight individuals (BMI < 18·5 kg/m^2^) and respondents with missing values for smoking, physical activity (PA), measured or self-reported height and measured weight. The analytical sample size for CCHS 2004 was 11 748 adults. Analyses of CCHS 2015 commenced in Fall 2017, after release and access of the data in June 2017. The same exclusion criteria were applied for a sample size of 12 110. The aforementioned sample sizes were used for all analyses of the likelihood of being affected by obesity across survey cycles.

For analyses presented in Tables 1–3 only, further exclusions included respondents missing the following socio-demographic variables: highest household education (below secondary education, secondary education, trade school/college or university), immigrant status (yes/no) and marital status (single, married or widowed), for a smaller sample size of 12 049. These results were not compared with CCHS 2004 data.

**Table 1:** Analysis of socio-demographic and lifestyle characteristics across quintile categories of DASH, DGAI 2015 and MSDPS scores among Canadian adults from CCHS 2015 (*n* 12 049)*

	DASH										DGAI 2015									
	Q1		Q2		Q3		Q4		Q5		Q1		Q2		Q3		Q4		Q5	
	Mean or %	se	Mean or %	se	Mean or %	se	Mean or %	se	Mean or %	se	Mean or %	se	Mean or %	se	Mean or %	se	Mean or %	se	Mean or %	se
Total mean score	27·5	0·2	37·6	0·1	44·3	0·1	51·3	0·1	61·9	0·2	6·1	0	7·7	0	8·8	0	10·1	0	11·9	0
Female, %	41	1·9	45·5	1·8	47·0	1·8	56·1	1·8	57·6	1·7	33·2	2·0	49·0	2·0	51·4	1·8	50·5	1·9	62·7	1·85
Age, years	45·9	0·6	47·8	0·7	48·7	0·6	49·9	0·6	52·3	0·6	45·2	0·7	47·5	0·6	48·9	0·6	50·9	0·7	52·2	0·5
BMI, kg/m^2^	28·7	0·2	28·3	0·2	27·7	0·2	27·6	0·2	27·2	0·2	28·6	0·2	28·3	0·3	27·6	0·2	28·0	0·2	27·0	0·2
Obesity, %	32·3	1·8	30·4	1·6	26·3	1·7	27·1	1·5	24·9	1·5	32·2	1·8	31·2	1·8	26·2	1·6	28·5	1·6	23·3	1·3
Current daily smokers, %	19·5	1·7	15·5	1·3	16·3	1·5	11·3	1·2	5·2	0·7	21·9	1·7	18·0	1·9	11·2	1·1	9·7	0·1	7	1
Met physical activity guidelines^||^,%	36·9	2	42·6	2·0	46·5	2·4	45·3	1·9	49·5	2·1	41·2	2·1	42·0	2·3	45·2	2·1	45·3	1·9	47·5	1·9^‡^
Highest household education, %																				
< Secondary school	10·2	0·8	8·3	0·7	7·3	0·6	6·0	0·5	4·7	0·5	10·8	0·8	8·5	0·7	6·3	0·6	6·3	0·5	4·6	0·4
Post-secondary education	31·4	1·9	37·7	1·7	40·7	1·9	45·8	2·0	52·7	2·1	31·0	1·8	36·7	1·9	44·7	2·0	44·6	2·1	53·0	1·9
Immigrant, %	23·8	2	25·8	1·9	24·5	1·9	28·9	1·9	34·1	2	21·0	1·8	20·4	1·8	28·9	2·1	29·8	2·0	36·7	2
Marital status, %																				
Single	23·3	1·7	22·0	1·4	20·7	1·6	20·7	1·6	16·9	1·3	27·4	2	23·8	1·6	18·0	1·4	18·0	1·2	16·7	1·2
Married	61·1	2·2	62·8	1·8	64·6	2·1	65·5	2·0	70·1	2·0^§^	55·7	2·3	60·3	2·1	68·3	1·9	68·8	2·0	70·2	1·8

	MSDPS									
	Q1		Q2		Q3		Q4		Q5	
	Mean or %	se	Mean or %	se	Mean or %	se	Mean or %	se	Mean or %	se
Total mean score	4·3	0·1	9·2	0·1	12·9	0·1	17	0·1	24·7	0·2
Female, %	46	1·8	50·7	1·8	48·3	1·9	50·7	1·8	52·9	1·9^†^
Age, years	48·2	0·6	46·8	0·7	48·9	0·6	49·1	0·6	51·5	0·5
BMI, kg/m^2^	28·2	0·2	28·0	0·2	27·6	0·2	27·9	0·2	27·7	0·2^¶^
Obesity, %	28·7	1·5	27·7	1·5	26·7	1·5	28·2	1·5	28·9	1·7**
Current daily smokers, %	20·6	1·7	13·4	1·3	15·4	1·7	9·6	1·0	8·2	1·1
Met physical activity guidelines^||^,%	42·4	2·2	43·1	2·1	42·8	2·1	47·4	2·1	46·1	2·2^††^
Highest household education, %										
< Secondary school	10·4	0·8	7·9	0·6	7·4	0·7	5·5	0·5	5·0	0·5
Post-secondary education	31·8	2·0	38·8	1·9	40·4	1·9	48·2	1·9	50·7	2·1
Immigrant, %	28·4	1·9	28·3	2·2	23·2	1·7	28·2	1·7	30·2	2·0^‡‡^
Marital status, %										
Single	24·8	1·6	23·0	1·6	20·1	1·4	17·9	1·2	17·3	1·3
Married	59·1	2·1	61·4	2·1	65·4	1·9	68·6	1·8	69·4	1·9

DASH, Dietary Approaches to Stop Hypertension^(16)^; DGAI, Dietary Guidelines for Americans Adherence Index^(10)^; MSDPS, Mediterranean-Style Dietary Pattern Score^(15)^; CCHS, Canadian Community Health Survey. Values are means or percentages ± standard errors (se). Estimates are weighted least squares means or percentages from a regression model adjusted for age and sex with bootstrapping to ensure accurate standard errors when using survey data. *P*_trends_ were estimated with the use of DASH, DGAI and MSDPS in their continuous form and represent the *P*-value associated with the linear regression coefficient for continuous variables and the logistic regression coefficient for categorical variables. All *P*_trends_ were < 0·0001 unless otherwise noted. Analyses were conducted on the first day of 24-dietary recall data only.

* Maximum possible scores for each diet quality index were: 90 out of 90 for DASH, 19 out of 19 for DGAI and 100 out of 100 for MSDPS.

† *P*_trend_ = 0·0368.

‡ *P*_trend_ = 0·0137.

§ *P*_trend_ = 0·0018.

|| Current Canadian physical activity guidelines for adults states reaching a goal of 150 min of moderate/vigorous-intensity physical activity per week.

¶ *P*_trend_ = 0·3394.

** *P*_trend_ = 0·8003.

†† *P*_trend_ = 0·1109.

‡‡ *P*_trend_ = 0·5025.

**Table 2:** Mean daily intake of macro- and micronutrients across quintile categories of DASH, DGAI 2015 and MSDPS scores among Canadian adults from CCHS 2015 (*n* 12 049)

	DASH										DGAI 2015									
	Q1		Q2		Q3		Q4		Q5		Q1		Q2		Q3		Q4		Q5	
	Mean or %	se	Mean or %	se	Mean or %	se	Mean or %	se	Mean or %	se	Mean or %	se	Mean or %	se	Mean or %	se	Mean or %	se	Mean or %	se
Energy intake, kcal	2283	24	2298	24	2300	23	2282	19	2249	20†	2350	26	2275	23	2267	20	2262	22	2249	20†
Energy density	2·1	0	2·0	0	1·9	0	1·8	0	1·5	0	2·3	0	2·0	0	1·8	0	1·7	0	1·4	0
Carbohydrate, % energy	42·9	0·5	45·0	0·4	46·0	0·5	48·3	0·4	51·3	0·4	41·9	0·4	46·3	0·5	47·3	0·5	48·9	0·4	50·2	0·4
Fibre density, g/1000 kcal	5·9	0·2	7·4	0·1	8·9	0·2	10·7	0·2	13·6	0·2	5·7	0·1	8·0	0·2	9·2	0·2	11	0·1	13·7	0·2
Total fat, % energy	36·0	0·4	34·3	0·4	33·2	0·5	31·7	0·4	30·0	0·4	36·9	0·4	34·1	0·5	32·4	0·4	31·3	0·4	29·6	0·3
SFA, % energy	12·3	0·2	11·7	0·2	10·9	0·2	10·1	0·2	8·6	0·1	13·0	0·2	11·3	0·2	10·7	0·2	9·7	0·1	8·5	0·1
MUFA, % energy	13·5	0·2	12·6	0·2	12·2	0·2	11·9	0·2	11·5	0·2	13·7	0·2	12·5	0·2	12·1	0·2	11·7	0·2	11·4	0·2
PUFA, % energy	7·1	0·2	6·8	0·1	7·1	0·3	6·8	0·2	7·1	0·1†	7·1	0·2	7·3	0·3	6·7	0·2	6·9	0·1	6·9	0·1†
Protein, % energy	18·4	0·3	17·1	0·2	16·6	0·3	16·2	0·2	15·5	0·2	15·8	0·2	15·8	0·2	16·7	0·3	17·1	0·3	18·5	0·2
Alcohol, % energy	2·6	0·3	3·7	0·3	4·3	0·3	3·9	0·3	3·2	0·3	5·4	0·4	3·8	0·3	3·7	0·3	2·8	0·3	1·8	0·2
Cholesterol density, mg/1000 kcal	204·1	7·2	162·3	4·3	139·5	4·3	127·9	4·4	99·2	4·4	179·5	6·8	147·7	4·4	144·9	5·3	124·6	3·8	125·4	5·1
Ca density, mg/1000 kcal	338·8	7·9	406·9	8·3	425·7	8·4	435·3	8·1	481·8	8·8	391·7	9·4	398·3	8·6	423·3	9·0	435	7·8	452·7	7·4
Vitamin A density in RAE, μg/1000 kcal	284·6	11·3	345·3	21·2	338·1	15·6	371·1	13·6	427·5	13·8	277·1	13·3	280·0	9·4	354·0	21·3	373·1	12·5	504·7	16
Vitamin D density, μg/1000 kcal	2·4	0·1	2·8	0·1	2·6	0·1	2·6	0·1	2·6	0·1†	2·4	0·1	2·4	0·1	2·6	0·1	2·8	0·1	2·9	0·1†
Vitamin C density, mg/1000 kcal	35·5	4	41·3	1·8	50·9	1·8	60·7	2·4	78·1	2·2	28·5	1·6	43·1	2·3	53·7	3·4	65·8	2·2	81·2	2·5
Na density, g/1000 kcal	1807	30	1534	25	1461	20	1324	20	1180	20	1527	23	1514	22	1500	28	1364	21	1348	20
Naturally occurring folate density, μg/1000 kcal*	88·1	4·8	95·8	1·8	113·0	2·5	126·6	2·3	157·7	3·4	84·3	1·9	98·7	2·6	116·1	4·4	128·2	2·3	162·3	3·2
Folacin density from food sources, μg/1000 kcal^‡^	153·7	4·8	165·4	2·7	171·9	2·8	183·4	3·0	202·8	3·4	149·7	2·5	165·0	3·0	178·9	4·7	183·8	2·7	206·1	3·0
P density, mg/1000 kcal	647·3	8·6	663·2	8·0	690·7	12·0	689·2	7·6	734·6	9·0	625·4	9·3	657·6	11·5	675·8	8·1	708·7	8·7	771·1	8·0
Mg density, mg/1000 kcal	134·0	4·2	144·3	1·8	160·1	2·0	180·0	2·3	213·2	2·7	130·1	2·7	153·9	4·1	164·1	2·3	181·0	1·7	212·1	2·5
Fe density, mg/1000 kcal	6·3	0·1	6·5	0·1	6·5	0.1	6·8	0·1	7·1	0·1	5·8	0·1	6·4	0·1	6·7	0·1	6·9	0·1	7·4	0·1
Zn density, mg/1000 kcal	5·8	0·1	5·5	0·1	5·5	0·1	5·4	0·1	5·7	0·1†	5·1	0·1	5·3	0·1	5·7	0·1	5·8	0·1	6·2	0·1
K density, mg/1000 kcal	1238	25	1335	16	1444	18	1571	17	1764	17	1168	20	1328	18	1480	21	1600	16	1851	18
Caffeine density, mg/1000 kcal	117·7	9·5	94·6	4·4	84·8	3·6	84·9	4·2	79·1	4·3†	104·8	5·5	109·2	9·4	84·9	4·3	82·8	4·0	74·2	4·1
Total energy from added sugars, %	10·4	0·4	8·5	0·3	7·1	0·3	6·5	0·3	5·4	0·2	9·7	0·4	9·1	0·4	7·4	0·3	6·6	0·3	4·4	0·2
Linoleic acid, % energy	6·0	0·1	5·7	0·1	6·1	0·3	5·8	0·1	6·1	0·1†	6·0	0·2	6·3	0·3	5·7	0·1	5·9	0·1	5·8	0·1
Linolenic acid, % energy	0·8	0	0·8	0	0,7	0	0·7	0	0·8	0†	0·8	0	0·7	0	0·7	0	0·8	0	0·8	0†
Glycaemic index	35·3	1·0	34·3	2·4	29·8	0·5	29·0	0·5	27·2	0·5	37·5	2·7	31·6	0·5	29·5	0·6	28·9	0·5	26·7	0·5

	MSDPS									
	Q1		Q2		Q3		Q4		Q5	
	Mean or %	se	Mean or %	se	Mean or %	se	Mean or %	se	Mean or %	se
Energy intake, kcal	2223	25	2244	23	2281	22	2313	21	2332	21†
Energy density	2·1	0	1·9	0	1·9	0	1·8	0	1·7	0†
Carbohydrate, % energy	48·4	0·5	48·8	0·5	46·4	0·5	46·1	0·4	44·9	0·4
Fibre density, g/1000 kcal	7·3	0·2	8·9	0·2	9·1	0·2	9·9	0·2	11·1	0·2
Total fat, % energy	32·3	0·4	32·0	0·4	34·1	0·5	33·1	0·4	33·2	0·3†
SFA, % energy	10·5	0·2	10·7	0·2	11	0·2	10·7	0·2	10·7	0·2†
MUFA, % energy	11·8	0·2	11·8	0·2	12·7	0·2	12·6	0·2	12·6	0·2
PUFA, % energy	6·9	0·2	6·8	0·2	7·3	0·2	6·9	0·1	7·0	0·2†
Protein, % energy	15·0	0·2	16·1	0·2	16·8	0·2	17·2	0·2	18·0	0·2
Alcohol, % energy	4·3	0·4	3·1	0·3	2·6	0·3	3·6	0·3	4·1	0·3
Cholesterol density, mg/1000 kcal	118·9	4·8	133	5·4	145·6	5·2	151·8	6·2	168·4	5·1†
Ca density, mg/1000 kcal	362·4	8·4	414·5	9·1	418·6	9·3	434·9	7·8	450·8	6·8
Vitamin A density in RAE, μg/1000 kcal	274·2	15·8	324·1	12·2	342·5	12·2	382·9	12·6	423·1	20·9
Vitamin D density, μg/1000 kcal	2·0	0·1	2·6	0·1	2·5	0·1	2·8	0·1	3·1	0·1†
Vitamin C density, mg/1000 kcal	32·0	2·1	52·2	3·5	51·9	2·1	57·0	2·1	68·6	2·6†
Na density, g/1000 kcal	1586	30	1508	26	1434	21	1423	21	1358	20†
Naturally occurring folate density, μg/1000 kcal*	89·0	2·7	112·2	4·2	111·6	2·4	122·0	2·6	139·9	3·3†
Folacin density from food sources, μg/1000 kcal^‡^	180·5		183·5	4·4	170·3	2·9	170·6	2·7	175·2	3·2†
P density, mg/1000 kcal	598·4	8·7	662·6	9·2	687·5	10·3	702·8	8·4	749·8	7·6†
Mg density, mg/1000 kcal	143·7	4	157·0	2·2	163·8	2·4	172·4	2·7	189·6	2·5†
Fe density, mg/1000 kcal	6·6	0·1	6·9	0·1	6·6	0·1	6·5	0·1	6·6	0·1†
Zn density, mg/1000 kcal	5·1	0·1	5·5	0·1	5·6	0·1	5·6	0·1	5·9	0·1†
K density, mg/1000 kcal	1182	21	1406	20	1445	17	1551	16	1700	19†
Caffeine density, mg/1000 kcal	117·3	9·9	94·8	4·5	84·0	3·8	80·8	4·1	86·2	4·0†
Total energy from added sugars, %	8·7	0·4	8·4	0·4	7·5	0·3	7·1	0·3	6·3	0·2
Linoleic acid, % energy	5·9	0·2	5·8	0·2	6·3	0·2	5·9	0·1	5·9	0·1†
Linolenic acid, % energy	0·8	0	0·7	0	0·8	0	0·8	0	0·7	0†
Glycaemic index	40·6	2·5	32·2	0·6	30·2	0·5	28·1	0·5	26·4	0·5

DASH, Dietary Approaches to Stop Hypertension; DGAI, Dietary Guidelines for Americans Adherence Index; MSDPS, Mediterranean-Style Dietary Pattern Score; CCHS, Canadian Community Health Survey; RAE, retinol activity equivalents. Values are means or percentages ± standard errors (se). Estimates are weighted least squares means or percentages from a regression model adjusted for age, sex and energy misreporting status (under-reporters, plausible reporters and over-reporters) with bootstrapping to ensure accurate standard errors when using survey data.

*P*_trends_ were estimated with the use of DASH, DGAI and MSDPS in their continuous form and represent the *P*-value associated with the linear regression coefficient.

Analyses were conducted on the first day of 24-dietary recall data only.

All *P*-_trends_ were < 0·0001 unless otherwise noted (†).

* Naturally occurring folate includes various forms of folate found naturally in food.

‡ Sum of quantities of naturally occurring folate in addition to folic acid without considering their differing bioavailability.

**Table 3:** Mean daily intake of select food groups and dietary components across quintile categories of DASH, DGAI 2015 and MSDPS scores among Canadian adults from CCHS-Nutrition 2015 (*n* 12 049)

	DASH										DGAI 2015									
	Q1		Q2		Q3		Q4		Q5		Q1		Q2		Q3		Q4		Q5	
	Mean or %	se	Mean or %	se	Mean or %	se	Mean or %	se	Mean or %	se	Mean or %	se	Mean or %	se	Mean or %	se	Mean or %	se	Mean or %	se
DGAI 2015 sub-components																				
‘Food Intake’ Sub-score’																				
Dark green vegetables, servings/week	1·4	0·2	1·6	0·1	1·9	0·1	2·4	0·2	3·2	0·2	0·8	0·1	1·2	0·1	19	0·2	2·7	0·2	4·2	0·2
Orange vegetables, servings/week	0·4	0·1	0·6	0·1	0·6	0·1	0·9	0·1	1·4	0·1	0·2	0	0·3	0·1	0·7	0·1	1	0·1	1·8	0·1
‘Other’ vegetables, servings/week ^1^	2·1	0·3	2·9	0·2	3·4	0·2	3·9	0·3	5·0	0·3	1·0	0·1	2·2	0·2	3·2	0·3	4·7	0·2	6·8	0·3
Starchy vegetables, servings/week	3·0	0·2	3·6	0·2	3·6	0·3	3·7	0·3	3·3	0·3	2·8	0·2	3·3	0·2	3·5	0·3	3·4	0·2	4·2	0·3†
Legumes and soy, servings/week	0·8	0·2	1·4	0·2	1·6	0·2	2·0	0·3	1·7	0·2	0·9	0·2	1·2	0·2	1·7	0·3	1·6	0·2	2·2	0·2
Whole fruit, servings/week	0·5	0	0·7	0	1·0	0	1·2	0	1·7	0·1	0·5	0	0·8	0·1	1·0	0·1	1·3	0·1	1·5	0·1
Fruit and vegetable variety	1·2	0	1·5	0	1·8	0	2·1	0	2·4	0	0·8	0	1·3	0	1·8	0	2·2	0	3·0	0
Meat and beans, servings/day	8·3	0·2	6·4	0·2	5·8	0·2	5·5	0·2	5·1	0·2	6·6	0·3	5·9	0·2	5·9	0·2	6·0	0·2	6·4	0·2†
Dairy, servings/day	1·0	0·1	1·4	0·1	1·6	0·1	1·6	0·1	1·7	0·1	1·5	0·1	1·4	0·1	1·5	0·1	1·5	0·1	1·6	0·1†
Total grains, servings/day	6·1	0·2	6·5	0·2	6·4	0·2	6·3	0·2	6·1	0·2†	5·9	0·2	6·5	0·2	6·5	0·2	6·4	0·2	6·1	0·2†
‘Healthy Choices’ Sub-score																				
Added sugar, % energy	10·4	0·4	8·5	0·3	7·1	0·3	6·5	0·3	5·4	0·2	9·7	0·4	9·1	0·4	7·4	0·3	6·6	0·3	4·4	0·2
Whole grains, % of total grains	5·5	0·7	10·3	1·0	17·4	1·1	22·3	1·2	37·0	1·3	6·1	0·8	13·0	1·0	16·8	1·1	25·0	1·1	35·7	1·3
Cholesterol intake, mg/d	436·8	21·5	358·1	11·0	318·8	12·1	291·9	11·6	242·5	12·0	411·0	20	329·5	11·2	321·7	12·4	283·2	11·2	279·6	13·1
Low-fat products, %	32·2	1·1	36·2	1·2	39·2	1·2	43·2	1·0	48·3	1·2	26·7	1·1	34·0	1·0	40·9	1·1	46·8	1·1	53·9	1·2
Na, mg/d	3814	62	3401	62	3248	52	3248	52	2717	50	3491	67	3297	50	3256	57	3026	56	2982	49
Alcohol, drinks/day	0·9	0·1	1·1	0·1	1·2	0·1	1·2	0·1	1·0	0·1†	1·6	0·1	1·1	0·1	1·1	0·1	0·9	0·1	0·7	0·1
DASH Sub-components																				
Total vegetable intake, servings/day	1·1	0·1	1·4	0	1·6	0·1	1·7	0·1	2·0	0·1	1·0	0	1·2	0	1·5	0·1	1·8	0·1	2·3	0·1
Plant protein intake, servings/day	0·3	0·1	0·7	0·1	1·2	0·1	2·0	0·1	2·8	0·1	0·7	0·1	1·2	0·1	1·4	0·1	1·7	0·1	2·2	0·1
Animal protein intake, servings/day	8·3	0·2	6·3	0·2	5·4	0·2	4·9	0·1	3·9	0·2	6·3	0·3	5·4	0·2	5·6	0·2	5·5	0·2	5·6	0·2†
MSDPS Sub-components																				
Fish and seafood, servings/week	2·4	0·2	1·9	0·2	1·6	0·2	1·4	0·2	1·4	0·2	1·7	0·2	1·5	0·2	1·6	0·2	1·9	0·2	1·8	0·2†
Red meat, servings/week	5·8	0·4	4·9	0·3	4·2	0·3	3·8	0·3	3·2	0·3	4·5	0·4	3·9	0·3	4·7	0·3	4·3	0·3	4·2	0·3†
Sweets, servings/week	15·9	1·2	15·5	0·9	13·2	0·8	12·6	0·8	11·3	0·8	14·7	1·1	15·7	0·9	13·3	0·8	13·4	0·8	10·8	0·8

	MSDPS									
	Q1		Q2		Q3		Q4		Q5	
DGAI 2015 sub-components	Mean or %	se	Mean or %	se	Mean or %	se	Mean or %	se	Mean or %	se
‘Food Intake’ Sub-score’										
Dark green vegetables, servings/week										
Orange vegetables, servings/week	1·3	0·2	1·8	0·1	2·3	0·1	3·0	0·2	3·0	0·2
‘Other’ vegetables, servings/week ^1^	0·4	0·1	0·7	0·1	0·7	0·1	0·9	0·1	1·1	0·1
Starchy vegetables, servings/week	2·0	0·2	3·0	0·2	3·5	0·2	3·7	0·2	4·8	0·3
Legumes and soy, servings/week	2·6	0·2	3·0	0·2	3·8	0·3	3·8	0·2	3·9	0·2
Whole fruit, servings/week	1·0	0·2	1·1	0·2	1·4	0·2	1·6	0·2	2·3	0·2
Fruit and vegetable variety	0·5	0	0·9	0·1	0·9	0·1	1·1	0·1	1·4	0·1
Meat and beans, servings/day	1·1	0·1	1·6	0	1·8	0	2·0	0	2·4	0
Dairy, servings/day	5	0·2	5·6	0·2	6·3	0·2	6·5	0·2	7·1	0·2
Total grains, servings/day	1·0	0·1	1·3	0·1	1·5	0·1	1·6	0·1	1·8	0·1
‘Healthy Choices’ Sub-score	7·3	0·2	6·9	0·2	6·4	0·2	5·9	0·2	5·2	0·2
Added sugar, % energy										
Whole grains, % of total grains	8·7	0·4	8·4	0·4	7·5	0·3	7·1	0·3	6·3	0·2
Cholesterol intake, mg/d	2·9	0·8	11·7	1·0	15·3	1·0	23·2	1·3	35·3	1·4
Low-fat products, %	272·2	13·1	297·4	12·7	321·7	12·8	350·2	20·8	377·0	12·2
Na, mg/d	27·0	1·1	36·2	1·2	39·0	1·1	45·5	1·2	48·0	1·0
Alcohol, drinks/day	3312	62	3226	58	3213	58	3247	57	3130	49†
DASH Sub-components	1·4	0·1	1·0	0·1	0·8	0·1	1·1	0·1	1·2	0·1
Total vegetable intake, servings/day										
Plant protein intake, servings/day	1·1	0·1	1·4	0·1	1·6	0·1	1·6	0·1	2·0	0·1
Animal protein intake, servings/day	0·6	0·1	1·0	0·1	1·5	0·2	1·7	0·1	2·1	0·1
MSDPS Sub-components	4·9	0·2	5·3	0·2	5·6	0·2	5·9	0·2	6·5	0·2
Fish and seafood, servings/week										
Red meat, servings/week	0·9	0·2	1·1	0·1	1·6	0·2	1·9	0·2	2·7	0·2
Sweets, servings/week	4·2	0·3	4·8	0·3	4·4	0·3	4·1	0·3	4·2	0·3†
	13·0	1·0	13·6	1·1	14·4	0·8	14·4	0·8	13·0	0·8†

DASH, Dietary Approaches to Stop Hypertension^1^; DGAI, Dietary Guidelines for Americans Adherence Index^2^; MSDPS, Mediterranean-Style Dietary Pattern Score^3^; CCHS, Canadian Community Health Survey. Values are means or percentages ± standard errors (se). Estimates are weighted least squares means or percentages from a regression model adjusted for age, sex and energy misreporting status (under-reporters, plausible reporters and over-reporters) with bootstrapping to ensure accurate standard errors when using survey data.

*P*_trends_ were estimated with the use of DASH, DGAI and MSDPS in their continuous form and represent the *P*-value associated with the linear regression coefficient.

All *P*_trends_ were < 0·0001 unless otherwise noted (†).

Analyses were conducted on the first day of 24-dietary recall data only.

### Physical activity and energy misreporting

In CCHS 2004, derived variables were provided for daily energy expenditure of respondents ≥ 12 years, from which respondents could be categorised into PA levels. In CCHS 2015, this was not provided. To categorise PA levels in 2015, respondents’ average PA per day in minutes was computed and cut-offs were applied to define respondents as either sedentary, low active, active or very active^(21)^. To preserve power and sample size, if individuals could not be categorised due to missing values for PA (*n* 48), levels were assigned according to Garriguet *et al*. (i.e. < 14 years was categorised as low active, ≥ 14 years was categorised as sedentary)^(22)^.

To account for energy misreporting, estimated energy requirements (EER) for each respondent were calculated using Institute of Medicine equations, which require an individual’s age, sex, PA level, height and weight^(21)^. To determine energy misreporting, respondents’ reported energy intakes from CCHS were compared with their calculated EER^(22–24)^. An energy intake < 70 % of the EER indicated under-reporting, and an energy intake > 1·42 of EER indicated over-reporting; those in between were considered plausible reporters^(22–24)^. In CCHS 2004 analyses, those whose EER could not be calculated were not included in analyses.

In CCHS 2015 analyses, if individuals self-reported their height and weight but did not consent to their height and weight being measured (*n* 2965), a correction factor was applied to estimate their BMI from their self-reported data, as determined by Statistics Canada^(25)^. This was done to preserve power and sample size. The USDA’s categorisation of individuals into energy levels in the 2015–2020 DGA was used to calculate EER for this subset of individuals, as the USDA specifications require only age, sex and PA level^(11)^ (see Appendix 1).

### Calculation of diet quality index scores

Three diet quality indexes were chosen for analysis in this study: the MSDPS measured adherence to a Mediterranean-type dietary pattern^(15)^, a DASH index developed by Matsunaga *et al*. measured adherence to a DASH-style dietary pattern^(16)^, and the 2015 DGAI measured adherence to a dietary pattern in-line with recommendations in the 2015–2020 DGA^(10)^. Each index’s scoring criteria can be found in Appendices 2–4.

### The Mediterranean-Style Dietary Pattern Score

The MSDPS developed by Rumawas *et al*. assesses a Mediterranean-type dietary pattern among Western populations^(15)^. Individuals were scored proportionally across thirteen dietary components commonly found in this dietary pattern; overconsumption received a penalty proportional to the amount consumed over the recommendation. Final scores were totalled out of 100 and weighted by a factor 0–1·0 representing the proportion of energy from Mediterranean diet-type foods. The range of possible scores was 0–100, with higher scores representing better adherence.

### The Dietary Guidelines for Americans Adherence Index

The 2015 DGAI measures adherence to the twelve energy-based USDA Food Patterns found in the 2015–2020 DGA^(10,11)^. The 2015 DGAI is divided into a ‘food intake’ sub-score with eleven components and a ‘healthy choices’ sub-score with eight components. Scoring was assigned proportionally with a penalty for overconsumption. The trans-fat component was omitted in this study due to a lack of trans fat data in CCHS. The range of possible scores was 0–19, with higher scores representing better adherence.

### Dietary Approaches to Stop Hypertension index^(16)^

The DASH index by Matsunaga *et al*. assesses adherence to a DASH-style dietary pattern, with scoring criteria modelled after the Healthy Eating Index 2010 to maintain a robust scoring algorithm^(16)^. Scores were assigned proportionally across nine dietary components using an energy density approach. The range of possible scores was 0–90, with higher total scores denoting better adherence.

### Statistical methods

All analyses were performed using SAS (version 9.4; SAS Institute Inc.). Analyses were age- and sex-adjusted and weighted using sample survey weights provided by Statistics Canada to ensure representative estimates. All standard errors were bootstrapped using the balanced repeated replication method with 500 replications to account for the complex sampling design used in CCHS^(17)^.

To assess the association between DASH, DGAI 2015 and MSDPS scores with lifestyle characteristics, least squares means were estimated across quintiles of each diet quality index and various socio-demographic variables, adjusting for age and sex in the analysis. To assess the association between scores and quality of the respective dietary patterns, least squares means were estimated across quintiles and selected macro- and micronutrients (to assess nutrient quality of the dietary patterns) and selected dietary components (to assess overall diet quality), adjusting for age, sex and energy misreporting status. Misreporting status in CCHS has been shown to adjust for implausible recalls and selective misreporting of healthy *v*. unhealthy foods in examining the association between dietary intakes and the likelihood of obesity and indirectly adjusts for socio-economic characteristics correlated with misreporting status, including education and smoking status^(24)^. Macro- and micronutrient intakes were reported either as a percentage of total energy or per 1000 kcal where appropriate. A *P*_trend_ < 0·0001 was considered significant.

Pearson correlation coefficients between pairs of indexes were calculated to examine the similarity between indexes. To evaluate concordance or agreement between pairs of indexes, total scores from each index were divided into quintiles, and the proportion of the sample falling into quintile categories for pairs of indexes was examined. These analyses were performed using 1 d of dietary recall from the CCHS 2015 sample only.

For examining the association between dietary patterns and the likelihood of being affected by obesity, both days of dietary recall were used, from both CCHS 2004 and CCHS 2015.

After calculating respondents’ index scores, the NCI method was used to produce total ‘usual’ index scores that were adjusted for age, sex, weekend/weekday and sequence of dietary recall (first/second)^(26,27)^. SAS macros for the NCI method are publicly available^(27)^. To use the NCI-adjusted index scores in logistic regression models with obesity prevalence as the outcome (binary yes/no, where ‘yes’ denotes BMI ≥ 30 kg/m^2^), scores were first regression calibrated using the SAS macro INDIVINT^(27,28)^. After input into the regression model, the beta estimate and its standard error were combined with the output from the DISTRIB macro to manually calculate OR and 95 % CI. To avoid misclassification of respondents, OR and 95 % CI were computed at the median of each quintile from the distribution of continuous total scores^(29)^.

In the logistic regression model with DGAI and obesity, covariates included age, sex (male/female), PA level (sedentary, low-active, moderately active and very active), smoking status (daily smokers, occasional smokers and non-smokers) and sequence of dietary recall analysed. In the regression model with DASH and obesity, alcohol was also adjusted for, as alcohol intake is not included in Matsunaga *et al*.’s DASH index^(16)^. Because MSDPS does not account for energy in its scoring criteria, the regression model with MSDPS and obesity was additionally adjusted for energy. To do this, energy intake was also estimated using the NCI method before entering it into the regression model to avoid the erroneous use of usual intake-estimated and single-day food intake variables in the same model. The NCI method univariate SAS macros were used for DGAI and DASH, while the NCI method bivariate SAS macros were used for MSDPS.

## Results

In CCHS 2004, estimated mean MSDPS, DASH and DGAI scores were 12·43 ± 0·14 out of 100, 44·18 ± 0·29 out of 90 and 8·82 ± 0·05 out of 19; in CCHS 2015, scores were 13·9 ± 0·13, 44·99 ± 0·25 and 8·99 ± 0·04, respectively. These mean scores translated to a roughly 12–14 % adherence to the recommendations in a Mediterranean style-dietary pattern, 49–50 % adherence to the recommendations in a DASH diet and 46–47 % adherence to the recommendations in the 2015–2020 DGA for both samples of CCHS 2004 and 2015.

### Socio-demographic and lifestyle variables in Canadian Community Health Survey-Nutrition 2015

Table 1 reports the socio-demographic and lifestyle characteristics for the sample of Canadian adults from CCHS 2015 (*n* 12 049) across quintile categories of DASH, DGAI 2015 and MSDPS scores using 1 d of recall. Compared with Q1, those in Q5 (i.e. higher index scores) were more likely to be older, non-smokers, married and with post-secondary education (*P*_trend_ < 0·0001 for all indexes). Except for MSDPS, those with the best adherence to a healthy dietary pattern were also more likely to be female, immigrants and less likely to be affected by obesity (*P*_trends_ < 0·0001 for DASH and DGAI 2015). Those who had the best adherence to DASH were also more likely to meet current Canadian PA guidelines (*P*_trend_ < 0·0001); however, the average percentage of respondents meeting PA guidelines in Q5 of DASH was low at 49·5 ± 2·1 %.

### Association of Dietary Approaches to Stop Hypertension, Dietary Guidelines for Americans Adherence Index 2015 and Mediterranean-Style Dietary Pattern Score with diet quality

Table 2 provides mean intake of specific macro- and micronutrients for the sample of Canadian adults from CCHS 2015 (*n* 12 049) for all indexes and using 1 d of recall. In general, compared with those in Q1, respondents in Q5 of DASH and DGAI 2015 (i.e. higher scores) were more likely to consume beneficial macro- and micronutrients (*P*_trends_ < 0·0001). Those most adherent to DASH and DGAI 2015 were more likely to consume less fat, cholesterol and Na and were less likely to consume an energy-dense diet (*P*_trend_ < 0·0001 for all). In general, there were fewer significant trends among macro- and micronutrient intake across quintile categories for MSDPS. Compared with Q1, respondents in Q5 consumed fewer carbohydrates and alcohol and greater intakes of fibre, protein, MUFA and Ca intake (*P*_trend_ < 0·0001 for all). For all indexes, those most adherent to the respective dietary pattern were less likely to consume energy from added sugars (*P*_trend_ < 0·0001 for all).

Focusing on the consumption of food groups (Table 3), in general, compared with Q1, respondents in Q5 for all three indexes were more likely to consume a variety of whole fruits and vegetables, more of their total grain intake as whole grains, more low-fat dairy and meat products, and overall had a greater total vegetable and plant protein intake (*P*_trend_ < 0·0001 for all). There was no significant trend across quintile categories for red meat or dairy intake in the DGAI 2015, or for red meat, Na or sweets intake for the MSDPS (*P*_trend_ > 0·0001 for all).

### Performance of Dietary Approaches to Stop Hypertension, Dietary Guidelines for Americans Adherence Index 2015 and Mediterranean-Style Dietary Pattern Score

Appendices 5–8 show the correlations between the three indexes as well as concordance plots. Moderate correlations were observed between MSDPS and DASH, and MSDPS and DGAI (*r* = 0·44 for both), with the highest correlation between DASH and DGAI (*r* = 0·69). All correlations were significant (*P* < 0·0001).

### Association between Dietary Approaches to Stop Hypertension, Dietary Guidelines for Americans Adherence Index 2015 and Mediterranean-Style Dietary Pattern Score with likelihood of being affected by obesity

**Fig. 1:**
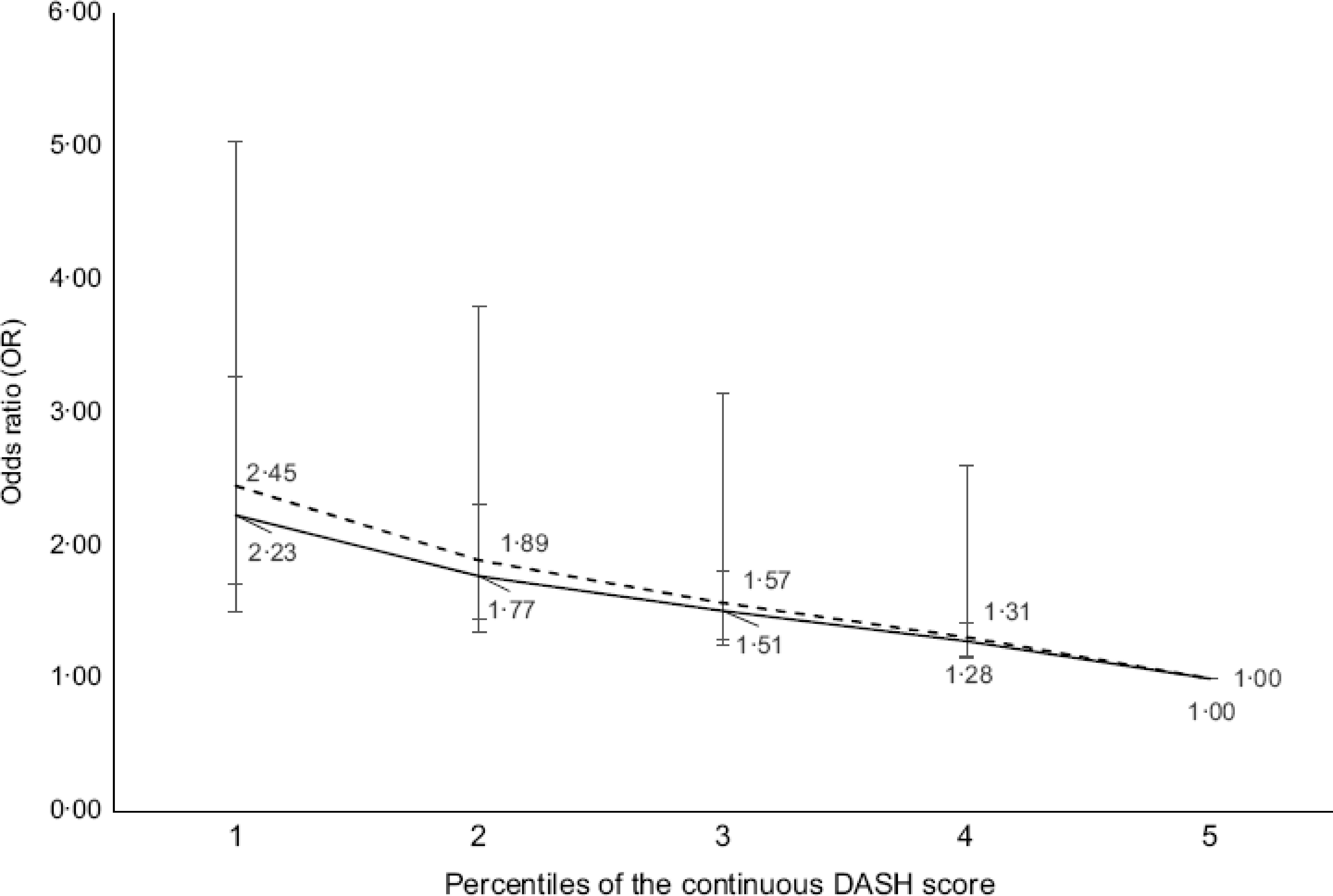
OR and 95 % confidence intervals for the association between the Dietary Approaches to Stop Hypertension (DASH) scores and likelihood of obesity (BMI ≥ 30 kg/m^2^) for the CCHS 2004 sample (*n* 11 748) and CCHS 2015 sample (*n* 12 110) of Canadian adults. CCHS 2004: dashed line CCHS 2015: solid line. The logistic regression model was adjusted for age, sex (male/female), physical activity level (sedentary, low-active, moderately active and very active), smoking status (daily smokers, occasional smokers and non-smokers), sequence of dietary recall analysed and alcohol intake; DASH scores were entered as continuous and the 90th percentile was used as reference. Analyses were conducted on both days of 24-dietary recall data

**Fig. 2:**
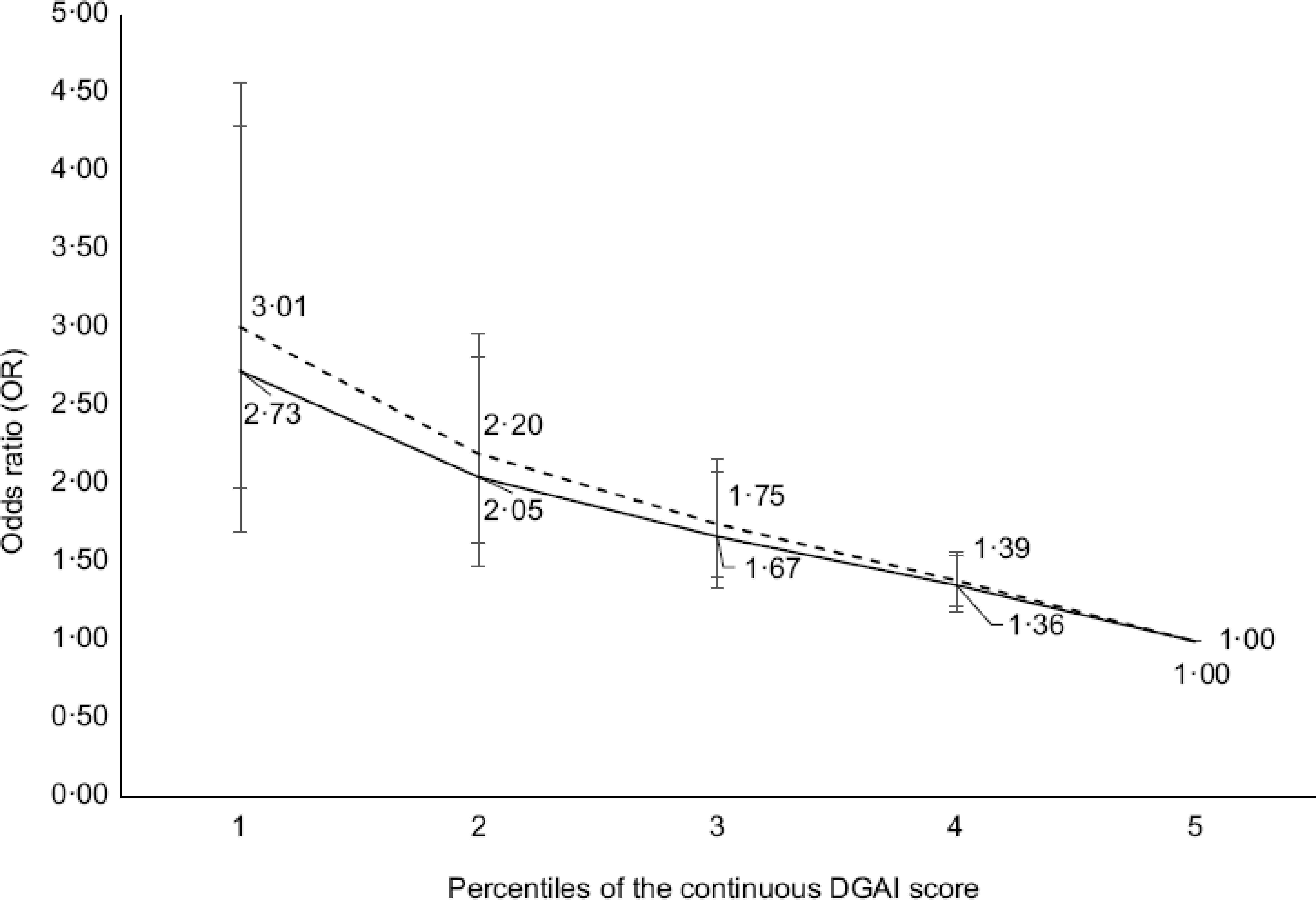
OR and 95 % confidence intervals for the association between Dietary Guidelines for Americans Adherence Index (DGAI) scores and likelihood of obesity (BMI ≥ 30 kg/m^2^) among a Canadian Community Health Survey (CCHS) 2004 sample (*n* 11 748) and CCHS 2015 sample (*n* 12 110) of Canadian adults. CCHS 2004: dashed line CCHS 2015: solid line. The logistic regression model was adjusted for age, sex (male/female), physical activity level (sedentary, low-active, moderately active and very active), smoking status (daily smokers, occasional smokers and non-smokers) and sequence of dietary recall analysed; DGAI scores were entered as continuous and the 90th percentile was used as reference. Analyses were conducted on both days of 24-dietary recall data

**Fig. 3:**
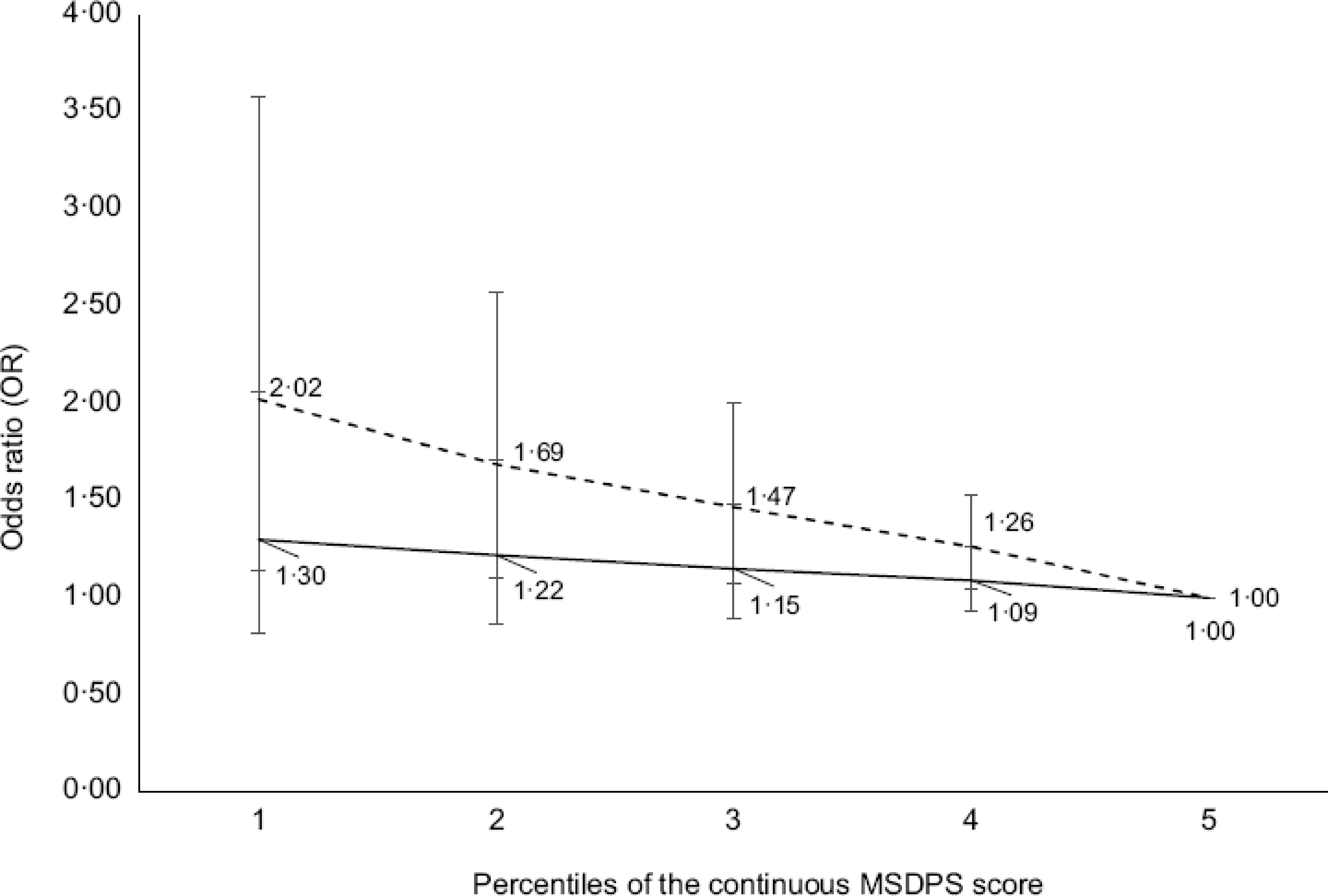
OR and 95 % confidence intervals for the association between Mediterranean-Style Dietary Pattern Scores (MSDPS) and likelihood of obesity (BMI ≥ 30 kg/m^2^) among a Canadian Community Health Survey (CCHS) 2004 sample (*n* 11 748) and CCHS 2015 sample (*n* 12 110) of Canadian adults. CCHS 2004: dashed line CCHS 2015: solid line. The logistic regression model was adjusted for age, sex (male/female), (under-reporters, plausible reporters and over-reporters), physical activity level (sedentary, low-active, moderately active and very active), smoking status (daily smokers, occasional smokers and non-smokers), sequence of dietary recall analysed and energy intake; MSDPS scores were entered as continuous and the 90th percentile was used as reference. Analyses were conducted on both days of 24-dietary recall data

Figures 1–3 illustrate the OR and 95 % CI for the likelihood of being affected by obesity at various percentiles of continuous index scores. Low scores for all indexes were associated with a greater likelihood of being affected by obesity in CCHS 2004 (dotted line; OR 10th *v*. 90th percentile and CI for DASH: 2·23 (95 % CI 1·50, 3·32), DGAI: 3·01 (95 % CI 1·98, 4·57) and MSDPS: 2·02 (95 % CI 1·14, 3·58)). Similar results were observed in CCHS 2015; however, results for MSDPS were not significant (solid line; OR 10th *v*. 90th percentile for MSDPS: 1·30 (95 % CI 0·82, 2·06)).

## Discussion

This is the first study to utilise two cycles of nationally representative Canadian health and nutrition survey data to assess the association between dietary patterns for chronic disease prevention and likelihood of being affected by obesity. Our results suggested that both a DASH-like diet and a diet in-line with the 2015–2020 DGA were associated with better diet quality and lower likelihood of obesity among Canadian adults, with an over 2·4-times greater likelihood of being affected by obesity when comparing those with weak adherence to those with best adherence to these dietary patterns. In contrast, our results indicated that a Mediterranean-type dietary pattern was not associated with obesity among the Canadian adult population.

Findings for obesity examined with DASH and DGAI 2015 have been echoed in the scientific literature^(4,7–10)^; however, results on the association between Mediterranean-type diets and obesity are mixed^(3–6,30)^. While some studies have reported reduced risks for those who are overweight or obese in those who follow a Mediterranean diet^(3–6)^, other studies have reported insignificant or null associations in European and East-Asian cohorts^(4,30)^. When looking at studies which specifically use the MSDPS to define a Mediterranean-type diet, a positive inverse association was reported between higher MSDPS scores and waist circumference in a study by the developers of the MSDPS^(15)^; however, no significant association was found between body weight or risk of obesity when it was applied to the SU.VI.MAX cohort^(4)^. Insignificant findings for MSDPS and the likelihood of being affected by obesity in this study were not completely unexpected and suggest poor discriminatory ability of MSDPS to distinguish between those with ‘healthier’ and ‘poorer’ diets. Furthermore, the mean total MSDPS score for both samples in this study ranged from 12 to 14 out of 100, suggesting extremely low adherence to this dietary pattern in the Canadian population. This may point to the impracticality of studying associations between Mediterranean-type diets and health outcomes using CCHS data because very few Canadians follow such a pattern. Dietary patterns for chronic disease prevention and management designed and tested in Canadian populations include the Portfolio diet^(31,32)^ (plant-based and focusing on cholesterol-lowering foods for cardiometabolic risk reduction) and the Prairie diet^(33)^ (diet pattern for diabetes management, designed to be in-line with Diabetes Canada’s clinical guidelines). Both the Portfolio and Prairie diets emphasise the use of healthy oils over animal fat; the Prairie diet also emphasises the consumption of dairy products. To study a Mediterranean-type diet in Canada, similar considerations for Canadian intakes (e.g. availability and preference for rapeseed oil instead of olive oil, and higher overall dairy intake) would have to be taken into consideration.

The methodology and reporting for this study were influenced by the Dietary Patterns Methods Project^(34)^. As was the case in Dietary Patterns Methods Project findings, results from our study suggested that there is no one approach to healthful eating for weight management. In our study, close adherence to all three dietary patterns was associated with a lower likelihood of obesity in both 2004 and 2015 cycles of CCHS data. Among the dietary patterns with the strongest association with obesity in both cycles (i.e. DASH and DGAI), those who ranked highly for adherence to DASH also ranked highly for adherence to DGAI (and vice versa), suggesting that a combination of beneficial dietary components, macronutrients and micronutrients may exist within different dietary patterns, while conferring possible benefits in weight management.

There are several strengths to this study. The use of two cycles of comprehensive, nationally representative data on Canadians’ intakes improved the generalisability and robustness of findings. Care was taken during methodological research design to reduce the effects of bias on results: the NCI method was used to estimate index scores in the context of ‘usual’ dietary intake, self-measured BMI was adjusted for using a validated correction factor to approximate measured BMI for those with missing measured height and weight data and all indexes were checked within the scientific literature for their validity, reliability and overall quality for application in this study. Despite these strengths, data from CCHS are cross-sectional and do not imply causal relationships. Residual confounding and reverse causation were also a possibility; however, the use of two cycles of CCHS data lowered the chances of this occurring. Additionally, BMI may not be the most precise marker of excess adiposity, especially for older adults who may lose height with age. However, as there are no additional markers of adiposity available in CCHS, our use of measured height and weight to examine obesity is justified.

While we were able to estimate index scores with the NCI method to capture some of the complexities due to long-term dietary intake, some within-person bias could not be accounted for in this study. This includes variation in individuals’ day-to-day intake and systematic choices in individuals’ preferences for combining foods (e.g. steak and potatoes). Additionally, the 24-h dietary recalls provided in CCHS 2015 were conducted by trained Statistics Canada interviewers and reviewed by dietitians; this may have introduced error into the collection of respondents’ dietary data.

In conclusion, the findings from this study of Canadian adults using comprehensive, nationally representative data suggested that, among Canadian adults, a DASH-like diet and a diet in-line with the 2015–2020 DGA may be associated with a lower likelihood of also being affected by obesity across two distinct time-points of data. These results aid in the understanding of diet and obesity in the Canadian context and highlight that multiple, distinct dietary patterns can be associated with weight management. Future prospective research is required to confirm these study findings.

## Supplementary material

For supplementary material accompanying this paper visit https://doi.org/10.1017/S1368980023000903. click here to view supplementary material
