# Supplementary material for: Adherence to a priori dietary patterns in relation to obesity: results from two cycles of the Canadian National Nutrition Survey
Source: Public Health Nutr. 2023 May 2;26(8):1596–608. doi: 10.1017/S1368980023000903 (PMC10410395; doi:10.1017/S1368980023000903)
Supplement: Supplementary file 1 [file S1368980023000903sup001.docx]

**Appendix 1**. Energy levels used for assignment of individuals to USDA Food Patterns in the 2015-2020 Dietary Guidelines for Americans. Modified from Table E3.1.A3 from the 2015-2020 Dietary Guidelines for Americans^1^.

|  | **Males** | |  |  | **Females** | | |
| --- | --- | --- | --- | --- | --- | --- | --- |
| **Age** | **Sedentary^1^** | **Moderately Active^2^** | **Active^3^** | **Age** | **Sedentary^1^** | **Moderately Active^2^** | **Active^3^** |
| 2 | 1000 | 1000 | 1000 | 2 | 1000 | 1000 | 1000 |
| 3 | 1000 | 1400 | 1400 | 3 | 1000 | 1200 | 1400 |
| 4 | 1200 | 1400 | 1600 | 4 | 1200 | 1400 | 1400 |
| 5 | 1200 | 1400 | 1600 | 5 | 1200 | 1400 | 1600 |
| 6 | 1400 | 1600 | 1800 | 6 | 1200 | 1400 | 1600 |
| 7 | 1400 | 1600 | 1800 | 7 | 1200 | 1600 | 1800 |
| 8 | 1400 | 1600 | 2000 | 8 | 1400 | 1600 | 1800 |
| 9 | 1600 | 1800 | 2000 | 9 | 1400 | 1600 | 1800 |
| 10 | 1600 | 1800 | 2200 | 10 | 1400 | 1800 | 2000 |
| 11 | 1800 | 2000 | 2200 | 11 | 1600 | 1800 | 2000 |
| 12 | 1800 | 2200 | 2400 | 12 | 1600 | 2000 | 2200 |
| 13 | 2000 | 2200 | 2600 | 13 | 1600 | 2000 | 2200 |
| 14 | 2000 | 2400 | 2800 | 14 | 1800 | 2000 | 2400 |
| 15 | 2200 | 2600 | 3000 | 15 | 1800 | 2000 | 2400 |
| 16 | 2400 | 2800 | 3200 | 16 | 1800 | 2000 | 2400 |
| 17 | 2400 | 2800 | 3200 | 17 | 1800 | 2000 | 2400 |
| 18 | 2400 | 2800 | 3200 | 18 | 1800 | 2000 | 2400 |
| 19-20 | 2600 | 2800 | 3000 | 19-20 | 2000 | 2200 | 2400 |
| 21-25 | 2400 | 2800 | 3000 | 21-25 | 2000 | 2200 | 2400 |
| 26-30 | 2400 | 2600 | 3000 | 26-30 | 1800 | 2000 | 2400 |
| 31-35 | 2400 | 2600 | 3000 | 31-35 | 1800 | 2000 | 2200 |
| 36-40 | 2400 | 2600 | 2800 | 36-40 | 1800 | 2000 | 2200 |
| 41-45 | 2200 | 2600 | 2800 | 41-45 | 1800 | 2000 | 2200 |
| 46-50 | 2200 | 2400 | 2800 | 46-50 | 1800 | 2000 | 2200 |
| 51-55 | 2200 | 2400 | 2800 | 51-55 | 1600 | 1800 | 2200 |
| 56-60 | 2200 | 2400 | 2600 | 56-60 | 1600 | 1800 | 2200 |
| 61-65 | 2000 | 2400 | 2600 | 61-65 | 1600 | 1800 | 2000 |
| 66-70 | 2000 | 2200 | 2600 | 66-70 | 1600 | 1800 | 2000 |
| 71-75 | 2000 | 2200 | 2600 | 71-75 | 1600 | 1800 | 2000 |
| 76 and up | 2000 | 2200 | 2400 | 76 and up | 1600 | 1800 | 2000 |

^1^Sedentary means a lifestyle that includes only the physical activity of independent living.

^2^Moderately Active means a lifestyle that includes physical activity equivalent to walking about 1.5 to 3 miles per day at 3 to 4 miles per hour, in addition to the activities of independent living.

^3^Active means a lifestyle that includes physical activity equivalent to walking more than 3 miles per day at 3 to 4 miles per hour, in addition to the activities of independent living.

**Appendix 2.** Scoring criteria for the Dietary Guidelines for Americans Adherence Index (DGAI) for individuals with 2000 kcal/day estimated energy requirement (EER)^2^

| **Components** | | | | | | | | | | | |
| --- | --- | --- | --- | --- | --- | --- | --- | --- | --- | --- | --- |
|  | | **Scoring Criteria** | |  | | | **Scoring Criteria** | | | |  |
|  |  | **0 Point** | **1.0 point** |  |  |  | **0 Point** | | **1.0 point** | |  |
| **Food Intake Sub-score** | |  |  | **Healthy Choice Sub-score** | | |  | |  | |  |
|  | Dark green vegetable (cups^1^/week) | 0 | ≥ 1.5 |  | Whole grain (% of grains) | 0 | | ≥ 50% | |  |  |
|  | Red/orange vegetables (cup/week) | 0 | ≥ 5.5 |  | Dietary fiber density (gram/1000kcal) | 0 | | ≥ 14 | |  |  |
|  | Legumes (cup/week) | 0 | ≥ 1.5 |  | Total fat (% Energy) | ≤ 10%, ≥ 45% | | ≥ 20%, ≤ 35% | |  |  |
|  | Starchy vegetables (cup/week)^2^ | 0 | 5.0 |  | Saturated fatty acid (% Energy) | ≥ 15% | | ≤ 10% | |  |  |
|  | Other vegetables (cup/week) | 0 | ≥ 4.0 |  | Cholesterol intake (mg/day) | ≥ 450 | | ≤ 300 | |  |  |
|  | Fruits (cup/day) | 0 | ≥ 2 |  | Low-fat dairy, and meat products (%) | 0% | | ≥ 75% | |  |  |
|  | Variety of fruits and vegetables (number of components) | 0 | 6.0 |  | Sodium (mg/day) | ≥ 3450 | | ≤ 2300 | |  |  |
|  | Grains (oz equivalent^1^/day)^2^ | 0 | 6.0 |  | Alcohol (drinks/day)^3^ | ≥ 1.5 | | ≤ 1.0 | |  |  |
|  | Meat and beans (oz equivalent/day)^2^ | 0 | 26 |  |  |  | |  | |  |  |
|  | Dairy (cup/day)^2^ | 0 | 3 |  |  |  | |  | |  |  |
|  | Added sugar (% Energy) | ≥ 9% | ≤ 6.0% |  |  |  | |  | |  |  |
|  | ^1^One cup is defined as 237 ml (US), 0.946 cup in metric unit; 1 oz=28.35 grams  ^2^An overconsumption penalty was imposed by reducing the score proportional to the amount of overconsumption up to 1.25 times higher than the recommended intake. Intakes ≥1.25 times the recommended amount were scored as 0.5.  ^3^One drink =118 ml wine;355 ml beer; or 45 ml distilled spirit | | | | | | | | |  |  |

**Appendix 3.** Scoring criteria for the Mediterranean-Style Dietary Pattern Score (MSDPS)^3^

| **Component** | **Standard for maximum score (10 points)^2^** | **Standard for minimum score (0 points)** |
| --- | --- | --- |
| Whole grains | ≥8 servings/day | 0 servings/day |
| Fruit | ≥3 servings/day | 0 servings/day |
| Vegetables | ≥6 servings/day | 0 servings/day |
| Dairy | ≥2 servings/day | 0 servings/day |
| Wine  Men  Women | 3 drinks/day  1.5 drinks/day | 0 drinks/day  0 drinks/day |
| Fish and other seafood | ≥6 servings/week | <6 servings/week |
| Poultry | ≥4 servings/week | <4 servings/week |
| Olives, legumes, and nuts | ≥4 servings/week | <4 servings/week |
| Potatoes and other starchy roots | ≥3 servings/week | <3 servings/week |
| Eggs | ≥3 servings/week | <3 servings/week |
| Sweets | ≥3 servings/week | <3 servings/week |
| Meat | ≥1 servings/week | <1 servings/week |
| Olive oil^1^ | Exclusive use | 0 points for no use of olive oil; 5 points for use with other vegetable oils |
| ^1^Except olive oil, all other components of the MSDPS were computed proportionately and a consumption penalty applied if intake was over the recommendation proportional to the number of servings exceeding the recommended intake.  ^2^The final total score (out of 130) was standardized to be out of 100; scores were then weighted by a factor 0-1 representing the proportion of energy coming from Mediterranean diet-type foods. | | |

**Appendix 4.** Scoring criteria for the Dietary Approaches to Stop Hypertension (DASH) index by Matsunaga et al.^4^

| **Component** | **Standard for maximum score (10 points)** | **Standard for minimum score (0 points)** |
| --- | --- | --- |
| Fruit | ≥1.2 cup equivalents^1^/1000kcal | 0 cup equivalents/1000kcal |
| Vegetables | ≥1.2 cup equivalents/1000kcal | 0 cup equivalents/1000kcal |
| Whole grains | ≥2.0 oz equivalents^1^/1000kcal | 0 oz equivalents/1000kcal |
| Dairy products | ≥1.6 cup equivalents/1000kcal | 0 cup equivalents/1000kcal |
| Plant proteins | ≥0.7 oz equivalents/1000kcal | 2 oz equivalents/1000kcal |
| Animal proteins | ≤2.4 oz equivalents/1000kcal | ≥4.5 oz equivlents/1000kcal |
| Added sugars | ≤2.3 tsp equivalents/1000kcal | ≥13.1 tsp equivalents/1000kcal |
| Sodium | ≤1095mg/1000kcal | ≥2099 mg/1000kcal |
| Saturated fat | ≤6% of total energy | ≥14% of total energy |
| ^1^One cup is defined as 237 ml (US), 0.946 cup in metric unit; 1 oz=28.35 grams | | |

| **Appendix 5.** Pearson correlation coefficients between DASH, DGAI and MSDPS, Canadian adults from CCHS 2015 (*n*=12,110). | | | |
| --- | --- | --- | --- |
|  | DASH | DGAI | MSDPS |
| DASH | 1.00 | 0.69 | 0.44 |
| DGAI |  | 1.00 | 0.44 |
| MSDPS |  |  | 1.00 |
| Analyses were conducted on the first day of 24-dietary recall data only. | | | |

|  | **Appendix 6.** Agreement or “concordance” between quintile combinations of computed DASH and DGAI scores in a sample of Canadian adults from CCHS 2015 (*n* = 12,110). | | | | | |
| --- | --- | --- | --- | --- | --- | --- |
|  |  | **Q1 (DASH)** | **Q2 (DASH)** | **Q3 (DASH)** | **Q4 (DASH)** | **Q5 (DASH)** |
|  | **Q1 (DGAI)** | 10 | 5.3 | 2.8 | 0.9 | 0.2 |
|  | **Q2 (DGAI)** | 5.5 | 5.6 | 4.9 | 2.8 | 0.8 |
|  | **Q3 (DGAI)** | 2.6 | 4.9 | 5.2 | 3.9 | 2.4 |
|  | **Q4 (DGAI)** | 1.2 | 2.6 | 5.5 | 5.8 | 6.1 |
|  | **Q5 (DGAI)** | 0.1 | 0.3 | 2.3 | 6.6 | 11 |
|  | Analyses were conducted on the first day of 24-dietary recall data only. Each cell displays the percentage (%) of the total sample falling into the respective quintile combinations, with darker shading representing a greater percentage of the sample. | | | | | |

| **Appendix 7.**  Agreement or “concordance” between quintile combinations of computed DGAI and MSDPS scores in a sample of Canadian adults from CCHS 2015 (*n* = 12,110). | | | | | |
| --- | --- | --- | --- | --- | --- |
|  | **Q1 (MSDPS)** | **Q2 (MSDPS)** | **Q3 (MSDPS)** | **Q4 (MSDPS)** | **Q5 (MSDPS)** |
| **Q1 (DGAI)** | 7.8 | 3.9 | 3.6 | 2.2 | 1.8 |
| **Q2 (DGAI)** | 5.0 | 4.3 | 4.7 | 2.6 | 2.5 |
| **Q3 (DGAI)** | 3.4 | 5.6 | 3.9 | 4.6 | 3.3 |
| **Q4 (DGAI)** | 2.0 | 4.0 | 4.5 | 4.3 | 5.3 |
| **Q5 (DGAI)** | 0.6 | 2.6 | 3.3 | 5.5 | 8.9 |
| Analyses were conducted on the first day of 24-dietary recall data only. Each cell includes the percentage (%) of the total sample falling into the respective quintile combinations, with darker shading representing a greater percentage of the sample. | | | | | |

| **Appendix 8.** Agreement or “concordance” between quintile combinations of computed DASH and MSDPS scores in a sample of Canadian adults from CCHS 2015 (*n* = 12,110). | | | | | |
| --- | --- | --- | --- | --- | --- |
|  | **Q1 (MSDPS)** | **Q2 (MSDPS)** | **Q3 (MSDPS)** | **Q4 (MSDPS)** | **Q5 (MSDPS)** |
| **Q1 (DASH)** | 7.4 | 4.5 | 3.6 | 2.4 | 1.2 |
| **Q2 (DASH)** | 5.1 | 4.6 | 4.3 | 3.0 | 2.5 |
| **Q3 (DASH)** | 3.5 | 3.8 | 4.3 | 4.1 | 3.3 |
| **Q4 (DASH)** | 2.0 | 4.4 | 4.2 | 4.7 | 6.0 |
| **Q5 (DASH)** | 0.7 | 3.0 | 3.7 | 5.0 | 8.8 |
| Analyses were conducted on the first day of 24-dietary recall data only. Each cell includes the percentage (%) of the total sample falling into the respective quintile combinations, with darker shading representing a greater percentage of the sample. | | | | | |

**REFERENCES**

1. United States Department of Agriculture. 2015-2020 dietary guidelines for Americans. <https://health.gov/dietaryguidelines/2015/>. Published 2015. Accessed September 18, 2017.
2. Jessri M, Lou WY, L'Abbe MR. The 2015 Dietary Guidelines for Americans is associated with a more nutrient-dense diet and a lower risk of obesity. *The American journal of clinical nutrition.* 2016;104(5):1378-1392.
3. Rumawas ME, Dwyer JT, McKeown NM, Meigs JB, Rogers G, Jacques PF. The development of the Mediterranean-style dietary pattern score and its application to the American diet in the Framingham Offspring Cohort. *The Journal of nutrition.* 2009;139(6):1150-1156.
4. Matsunaga M, Hurwitz EL, Li D. Development and Evaluation of a Dietary Approaches to Stop Hypertension Dietary Index with Calorie-Based Standards in Equivalent Units: A Cross-Sectional Study with 24-Hour Dietary Recalls from Adult Participants in the National Health and Nutrition Examination Survey 2007-2010. *Journal of the Academy of Nutrition and Dietetics.* 2018;118(1):62-73.e64.
